# Vaginal deployment and tenofovir delivery by microbicide gels

**DOI:** 10.1007/s13346-015-0227-1

**Published:** 2015-04-15

**Authors:** Y. Gao, A. Yuan, O. Chuchuen, A. Ham, K. H. Yang, D. F. Katz

**Affiliations:** Department of Biomedical Engineering, Duke University, Room 136 Hudson Hall, Box 90281, Durham, NC 27708 USA; ImQuest Biosciences, Frederick, MD 21704 USA; Eshleman School of Pharmacy, University of North Carolina, Chapel Hill, NC 27599 USA; Department of Obstetrics and Gynecology, Duke University Medical Center, Durham, NC 27710 USA

**Keywords:** Microbicide, Compartmental model, Pharmacokinetics, Pharmacodynamics, Gel, Vagina, Tenofovir

## Abstract

Gels are one of the soft material platforms being evaluated to deliver topically acting anti-HIV drugs (microbicides) to the vaginal environment. For each drug, its loaded concentration, gel properties and applied volume, and frequency of dosing can be designed to optimize PK and, thence, PD. These factors also impact user sensory perceptions and acceptability. Deterministic compartmental modeling of vaginal deployment and drug delivery achieved by test gels can help delineate how multiple parameters characterizing drug, vehicle, vaginal environment, and dosing govern details of PK and PD and also gel leakage from the canal. Such microbicide delivery is a transport process combining convection, e.g., from gel spreading along the vaginal canal, with drug diffusion in multiple compartments, including gel, mucosal epithelium, and stroma. The present work builds upon prior models of gel coating flows and drug diffusion (without convection) in the vaginal environment. It combines and extends these initial approaches in several key ways, including: (1) linking convective drug transport due to gel spreading with drug diffusion and (2) accounting for natural variations in dimensions of the canal and the site of gel placement therein. Results are obtained for a leading microbicide drug, tenofovir, delivered by three prototype microbicide gels, with a range of rheological properties. The model includes phosphorylation of tenofovir to tenofovir diphosphate (which manifests reverse transcriptase activity in host cells), the stromal concentration distributions of which are related to reference prophylactic values against HIV. This yields a computed summary measure related to gel protection (“percent protected”). Analyses illustrate tradeoffs amongst gel properties, drug loading, volume and site of placement, and vaginal dimensions, in the time and space history of gel distribution and tenofovir transport to sites of its anti-HIV action and concentrations and potential prophylactic actions of tenofovir diphosphate therein.

**Electronic supplementary material:**

The online version of this article (doi:10.1007/s13346-015-0227-1) contains supplementary material, which is available to authorized users.

## Introduction

Products for delivery of topically acting anti-HIV drugs (microbicides) are important alternatives to vaccines in prevention strategies against sexually transmitted HIV. Both the vaginal and rectal environments are being targeted [1–3]. Multiple microbicide drugs are being evaluated and are formulated in several types of vaginal delivery systems, including gels, rings, films, woven fiber meshes, and foaming tablets [2, 4–7]. These drugs act against HIV by several mechanisms of action within vaginal fluids and/or the mucosal tissue that contains host cells which sexually transmitted HIV can infect. Clearly, the mission of a microbicide product is to establish concentration distributions, in time and space, of its active drug(s) in target fluids and/or tissues, which are sufficient to prevent infection.

Vaginal gels have received much attention as microbicide delivery systems. However, no gel, to date, has successfully completed phase 3 trials. Most recently, a gel containing the antiretroviral drug tenofovir showed efficacy in its first phase 3 trial (CAPRISA 004) but failed in its second and third trials (VOICE, FACTS) [8–10]. The latter two failures have been attributed in large part to very poor trial participant adherence to specified gel dosage regimen. Clearly, microbicide gel success derives from synergy of efficacious pharmacokinetics (PK) and pharmacodynamics (PD) as linked to adequate willingness to use. The CAPRISA 004 trial showed that these could be achieved for the tenofovir gel loaded with 1 % drug and applied in a 4-mL volume in a before-and-after-sex (BAT24) regimen. Results from subsets of participants in the VOICE and FACTS trials who did consistently use the gel also suggested moderate efficacy. However, the VOICE and FACTS trials showed that adequate willingness to use was not achieved for this gel-volume combination, either in once daily (VOICE) or on-demand BAT24 (FACTS) dosing.

Microbicide PK derives from the interaction of gel spreading along the vaginal canal and drug transport out from the gel and into ambient fluid(s) and the layers of the vaginal mucosa. Gel spreading and drug mass transport relate to drug properties and loading, gel properties and volume, frequency of gel application, and properties of the host environment, including sexual activity and semen [3]. Willingness to use derives in part from user sensory perceptions and preferences of a gel in relation to its dosage regimen. Those perceptions relate in part to gel spreading along the vaginal canal and leakage therefrom, which derive from properties of the gel and its applied volume [11, 12]. Thus, there is an overlap in the influence of gel spreading and thus gel properties and volume upon pharmacological prophylactic efficacy and behavioral willingness to use. Deterministic modeling of gel deployment and drug delivery is a scientific tool that can help understand the determinants of both the PK and user willingness to use. Such modeling can thus contribute to objective design of microbicide gels that succeed both pharmacologically and behaviorally.

Tenofovir (TFV) exhibits its anti-HIV function (reverse transcriptase inhibition) after entering HIV-infectible host cells and becoming phosphorylated to tenofovir diphosphate (TFV-DP). The lower of the two vaginal mucosal layers, the stroma, contains a population of these cells and is believed to be a primary site of infection by vaginally transmitted HIV. Contemporary pharmacokinetic modeling of microbicide drug products (e.g., population PK models) has utilized empirical approaches in which each compartment is homogeneous, and the drug transport process is highly simplified to first-order exchange between compartments [13]. These approaches are quite valuable to our understanding of whole body microbicide PK and are helping quantify sources of variability in it. However, by nature, they do not capture all physical and physicochemical mechanisms that drive drug mass transport. Such transport is governed by convection and diffusion processes, which depend upon rates of gel spreading and concentration gradients within compartments. Consequently, the empirical models cannot objectively account for the non-linear multiparametric dependence of drug concentration distributions upon characteristics of product, its dosing, and host environment nor can they characterize spatial concentration distributions of drugs within compartments (which can be quite non-uniform [14], see below). The present study builds upon earlier modeling work by our group in initiating creation and application of a deterministic methodology to contribute to understanding the determinants of the drug transport process. In doing so, we apply a modeling approach that has been developed and proven beneficial in many other drug delivery contexts, e.g., transdermal delivery [15, 16].

Drug release from a gel into the vaginal mucosa occurs over the contact (coating) area of gel and epithelial surface. Drug is also released from gel into ambient vaginal fluids and, subsequently, may enter the mucosa. The gel-mucosal contact surface grows over time after gel insertion. Initial analyses of the mechanisms of microbicide gel drug delivery performance drew inferences from gel spreading (i.e., the evolving contact surface area) per se since this is a major factor in delivery. A succession of fluid mechanical models was developed [17–23]. A semi-empirical algorithm for drawing inferences about drug delivery from the original gel spreading model was developed and applied [24–26]. Recently, we introduced the first deterministic, computational model of mass transport by a drug from a vaginal gel into the vaginal mucosal tissue [14]. Here, the geometry of the processes was simplified: There was complete gel coating of the surfaces of the vaginal canal at a constant thickness. The model was applied quantitatively to the tenofovir gel that is currently in extended clinical testing, and good agreement was found between predictions of it and human PK data (see “Discussion”). However, that model was limited in that it computed only tenofovir concentrations, not those of its bioactive form, tenofovir diphosphate. Further, this model cannot capture the effects of time-dependent gel spreading along the vaginal canal and possible leakage from the vaginal opening, the introitus. Consequently, the model cannot distinguish consequences of spreading by gels with different properties and volumes in canals with different dimensions and of different locations of gel insertion along the canal. Indeed, vaginal gel spreading models, to date, have neglected these latter factors and have used a bilaterally symmetrical spreading model along a canal of infinite extent.

The present analysis is the logical next step in development and application of models of vaginal gel deployment and drug delivery into and throughout the vaginal environment. First, we introduce improvements in the theory of drug mass transport within and between compartments, accounting for gel spreading and leakage. Then, we apply the new theory to three prototype microbicide gels with contrasting rheological properties and consider variations in gel volume and drug loading. In doing so, we address the consequences of salient factors in gel use, including varying vaginal size and site of gel insertion within the canal. We contrast vaginal spreading and gel leakage vs. time for the three test gels. These results are input to computations of the time and space histories of tenofovir and tenofovir diphosphate concentration distributions. Such predictions can and are compared to direct experimental measurements in studies of drug pharmacokinetics. We also begin to interpret predicted tenofovir diphosphate concentration distributions with respect to target concentrations believed to be prophylactic against HIV. On the basis of an EC50 value for TFV-DP in cells against HIV, we compute a measure of TFV-DP concentration distribution in vaginal stroma that may be related to potential gel prophylactic efficacy (PD). Termed “percent protected,” this is a summary metric of gel product tenofovir delivery performance. It is a function of time and provides insights about the length the interval following gel insertion after which maximum protection is created, the magnitude of that protection, and its duration. These results can thus inform instructions to gel users about the safe interval of protection between gel insertion and sex. They can complement information derived from experimental PK studies which, by nature, are limited in the numbers of samples and sample times that can be implemented and in which data are beset by high variability [27, 28]. Together with the computations of gel leakage, they can guide decisions about optimal combinations of volume and drug loading for gels with particular rheological properties. Overall, our goal is to introduce a computational tool that can be used in the design and performance evaluation of current microbicide products and in the development of improved ones.

## Materials and methods

Drug delivery in the vaginal mucosa by a gel placed into the canal derives from two interacting mass transport processes: gel flow after insertion due primarily to squeezing by the walls of the canal [22] and drug diffusion out from gel and into tissue. Gel flow is essentially independent of drug transport and, thus, can be analyzed first and then input to solution of the complete convection–diffusion drug transport problem.

**Fig. 1:**
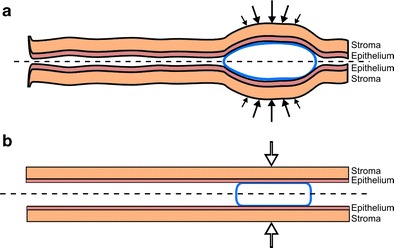
Geometrical features of the gel flow and drug transport problem, showing the epithelial and stromal layers of the mucus with a gel bolus inserted in between.** a** Physically realistic model; the *arrows* indicate variable squeezing force that is proportional to vaginal wall displacement. **b** Our rectilinear model geometry that approximates this; the *arrows* indicate total squeezing force from the vaginal wall. The problem is symmetrical about *y* = 0 (*dotted line*). For the human, a typical length of the vaginal canal is 13 cm and width is 3.35 cm. Typical thicknesses of epithelial and stromal tissue of the mucosa are 200 μm and 2.8 mm respectively (details are discussed in text)

**Fig. 2:**
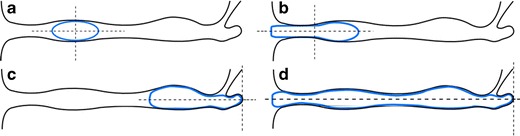
Four different cases of the extent of gel flow over the time interval of interest for different locations of initial gel bolus placement. The introitus of the vagina is the opening on the left side of the figure; the cervical canal inserts at an angle at the right. Gel profile is shown in *blue*. **a** Gel is initially placed in the canal; flow is bilaterally symmetric. **b** Gel has begun to leak; the flow is symmetric about midpoint of the gel in the vagina. **c** Gel has reached the end of the fornix; flow is towards the introitus only. **d** A combination of **b** and **c**, where the gel has coated the entire length of the canal

Here, we introduce improvements to prior models of vaginal gel spreading. Characterization of some geometric details of the human vaginal canal is simplified to facilitate some aspects of model implementation and interpretation. The simplifications are sufficient to enable a model to capture the essential physics of its transport process, while enabling initial comparisons with experimental results (see “Discussion”). The human vaginal canal has a cross-section that is approximately rectangular, with height small compared to width (typically about 2.5 cm), which in turn is small vs. axial length (ranging from about 10–15 cm [29, 30]). The internal cervical os inserts into the vaginal fornix at an oblique angle. The cervical canal is filled with mucus that constrains gel flow from the fornix into it. Both the outer opening (the introitus) and opening in the innermost wall of the vaginal canal connect to larger spaces: the introitus to the outside of the body and the os in the fornix to the upper female reproductive tract and peritoneal cavity. We assume that ambient pressure at the introitus and external cervical os is relatively constant and small compared to the time-dependent pressure within the canal, which is increased due to squeezing by the vaginal walls. Fluid mechanical lubrication theory can be applied for vaginal gel flow [17, 18], and the geometry can be reduced to a two-dimensional rectangular channel [18, 22]. We neglect variations across the width of the cross-section (the third dimension) and assume that pressure at the introitus is atmospheric. Gel is typically inserted as a bolus by a piston-type applicator. The bolus distends the canal walls, and consistent with the squeezing channel geometry, we assume that the shape of the bolus is rectangular and that it fills the height of the canal (Fig. 1) [22]. Initial gel placement influences its flow over time because (1) gel may spread to an extent that its outermost edge leaks from the introitus and/or (2) the innermost edge of gel may flow up to the inner wall of the fornix and thence cease to move. In our analysis of gel spreading, we use a Cartesian coordinate system with origin (*y* = 0) in the midplane of the gel and canal. Anatomically, the longitudinal position of the origin (*x* = 0) depends upon site of gel insertion (Fig. 2). Flow and drug transport are symmetrical about *y* = 0. The microbicides field has experimented with gel volumes ranging from 2 to 5 mL, with a 5-mL gel showing significant leakage. In our analyses, we picked gel volumes of 2 and 4 mL as representative values that span the likely range of volumes of future products.

### Analysis of gel flow along the lumen of the vaginal canal

Given that the site of initial gel placement can vary along the length of the canal, we define four configurations of flow that span the range of geometric possibilities. These differ in relation to whether or not the distal gel edge (moving outwards towards the introitus) and proximal edge (moving inwards towards the inner boundary of the fornix) reach those boundaries during the time course of flow (Fig. 2). If the distal edge reaches the introitus, the gel will leak out of the flow domain thereafter. If the proximal edge reaches the inner boundary of the fornix, subsequent gel flow will be unidirectional towards the introitus. Depending upon the parameters of the problem (gel rheology and volume and vaginal dimensions), the overall flow during the time course of interest may be a sequence of two or more of these cases.

Spreading of gel along the canal is a biomechanical flow problem for a non-Newtonian fluid in a channel with elastic walls that exhibit a squeezing force against it [17–20]. Mathematical details of our solution of this problem are in the Supplementary Material section. In this analysis, we characterize the rheological behavior of the gel using the Carreau-like constitutive model, which relates local shear stress in the gel to local shear strain rate [18]. In doing so, we include the possible presence of a yield stress in the gel. This constitutive equation is preferable to the Power Law constitutive model used in the initial modeling of vaginal gel flow [17, 31] because it more accurately accounts for the low shear strain rate behavior of the gel, which is characteristic of vaginal coating flow and which plays a major role in governing flow rate.

We applied this modeling schema to three gels that were created with rheological properties that bracket a range of those postulated to be candidates for vaginal microbicide gels, producing a range of spreading rates along the canal. This provided contrasts in how tenofovir delivery was influenced by gel rheology as well as other factors. Additional physicochemical gel properties (e.g., osmolality and pH) were not tuned to final values suitable for in vivo gel evaluations. Gel compositions are given in Table 1.

**Table 1:** Compositions and properties of the three test gels

	HEC (%)	Glycerol (%)	Methyl paraben (%)	Propyl paraben (%)	Phosphate buffer (%)	Osmolality (mOsm/kg)	pH
DG-01	3.00	2.50	0.20	0.05	93.25	360 ± 16	5.78
DG-02	4.00	2.00	0.20	0.05	92.75	355 ± 14	5.82
DG-03	1.50	2.00	0.20	0.05	95.25	331 ± 41	5.58

*HEC* hydroxyethylcellulose

Gel rheological properties were measured at body temperature (37 °C) using a constant stress protocol on a TA Instruments model AR 1500ex rheometer (4° cone and 20-cm plate configuration [19, 20]). Shear rates ranged 10^−4^ to 250 s^−1^. Residual stresses of gels were measured, as surrogates for yield stress by stress relaxation experiments in a Brookfield 5HB DV-III Ultra rheometer with a CPE-40 cone [19, 32]. The gel was initially stressed at 10 s^−1^ for 5 min and then relaxed for 14 min, during which time stress was measured to determine a limiting value. Results are given in Table 2.

**Table 2:** Rheological parameters of the three test gels based on the Carreau-like constitutive model

Gel	Yield stress *τ*_0_ (dyne/cm^2^)	Zero shear viscosity *m*_0_ (poise)	Flow consistency index *m* (dyne/cm^2^·s^n^)	Shear thinning index *n*
DG-01	20	3820	1300	0.261
DG-02	380	2070	1960	0.219
DG-03	0	175	153	0.344

### Drug transport into epithelial and stromal layers of the vaginal mucosa

Drug transport from gel into the vaginal mucosa is characterized in our model by a two-dimensional unsteady convection–diffusion mass transport process. The vaginal mucosa consists of two histologically different layers, the epithelium and stroma (Fig. 1). In humans, the upper epithelial layer has a stratified squamous cell structure, but no vasculature. The stromal layer is made up of connective tissue and some cells (a subset of which are infectible by HIV) and contains blood and lymphatic vessels which drug enters. Our model contains four different compartments, in each of which conservation of mass is applied: gel, epithelium, stroma, and the blood (which is taken as a spatially homogeneous compartment [28, 14, 27]). Drug transport is due to diffusion (in gel, epithelium, and stroma) and convection (in gel and vaginal fluid adjacent to it). Vaginal fluid is not taken as a separate compartment, but gel dilution by it is included in modeling mass transport in/out from the gel. The diffusion coefficient of drug in the gel is several orders of magnitude higher than the diffusion coefficient(s) in the tissue compartments [14]. We found, consequently, in our prior one-dimensional model that the time-dependent drug concentration in the gel was nearly uniform with depth, and we make that assumption here. Drug transport in both the epithelium and stroma remains a two-dimensional diffusion-driven process. Tenofovir diphosphate production is included in the epithelial and stromal compartments, which contain cells that tenofovir enters and become phosphorylated. The stroma contains HIV-infectible host cells that are believed to be a primary source of HIV transmission. Thus, the TFV-DP concentration in the stromal host cells is a primary output of the model. The conservation of mass equations for tenofovir and tenofovir diphosphate are as follows:

9a $$ \frac{d{C}_G}{dt}={D}_E\frac{4w}{V_G}{\displaystyle \underset{x=0}{\overset{L}{\int }}{\left.\frac{\partial {C}_E}{\partial y}\right|}_{y=0}}dx-{k}_D{C}_G $$

9b $$ \frac{\partial {C}_E}{\partial t}={D}_E\left(\frac{\partial^2{C}_E}{\partial {x}^2}+\frac{\partial^2{C}_E}{\partial {y}^2}\right)-{k}_{\mathrm{on}}\left\{{C}_E{\varphi}_E-\frac{C_{DP}}{r}\right\}+{k}_{\mathrm{off}}{C}_{DP} $$

9c $$ \frac{\partial {C}_S}{\partial t}={D}_S\left(\frac{\partial^2{C}_S}{\partial {x}^2}+\frac{\partial^2{C}_S}{\partial {y}^2}\right)-{k}_B{C}_S-{k}_{\mathrm{on}}\left\{{C}_S{\varphi}_S-\frac{C_{DP}}{r}\right\}+{k}_{\mathrm{off}}{C}_{DP} $$

9d $$ {V}_B\frac{d{C}_B}{dt}={\displaystyle \underset{0}{\overset{h_s}{\int }}{\displaystyle \underset{0}{\overset{d}{\int }}{k}_B{C}_S} dxdy}-{k}_L{C}_B $$

9e $$ \frac{\partial {C}_{\mathrm{DP}}}{\partial t}={k}_{\mathrm{on}}\left\{{C}_{\mathrm{TFV}}\varphi -\frac{C_{\mathrm{DP}}}{r}\right\}-{k}_{\mathrm{off}}{C}_{DP} $$

Here, concentrations are defined as per unit volume of the matrix (viz. the four compartments). Equation (9a) gives change in concentration *C*_*G*_ in the gel compartment, where *D*_*E*_ is the diffusion coefficient in the epithelium and *C*_*E*_ is concentration in the epithelium, *w* is the width of the canal, *L* is the distance from the center to the edge of the gel, and *V*_*G*_ is the gel volume. The integral gives total mass of drug leaving the gel to the epithelium. Drug concentration in gel is also reduced due to imbibing of ambient vaginal fluid, and this is modeled as a first-order process with rate constant *k*_*D*_ [14]. Drug transport in epithelium (Eq. (9b)) is a two-dimensional unsteady diffusion process, *C*_*E*_ is concentration, and *D*_*E*_ is the diffusion coefficient. The last two terms of the equation are the creation and elimination rate for tenofovir diphosphate, where *k*_on_ is the formation rate of TFV-DP, *k*_off_ is the elimination of TFV-DP, *φ*_*E*_ is the volume fraction of cells in the epithelium, and *r* is the fraction of TFV converted to TFV-DP within the cells. Drug transport in stroma (Eq. (9c)) is also a two-dimensional unsteady diffusion process with a first-order loss term for uptake into the vasculature with rate constant *k*_*B*_ [14]; *C*_*s*_ is concentration, and *D*_*S*_ is the diffusion coefficient. The TFV-DP production mechanism is taken to be similar to the one in the epithelium except with a different host cell volume fraction *φ*_*S*_. The blood vessels are distributed relatively uniformly throughout the stromal layer. In the blood compartment (Eq. (9d)), the mass balance of concentration *C*_*B*_ is governed by the input from the stroma divided by *V*_*B*_ (the volume of the blood compartment) and loss due to metabolism by the body (with first order rate constant *k*_*L*_, typically given as a volumetric value [33]). The time rate of change for TFV-DP (Eq. (9e)) has two components on the right hand side of the equation. The first is the rate of formation *k*_on_ multiplied by the difference between the current level of TFV-DP and the saturation level based on the local tenofovir concentration *C*_TFV_ in the epithelium or stroma, the proportion of host cells *φ*, and the fraction *r* of TFV that can be converted to TFV-DP. This term is inside Macaulay brackets {} (defined such that the expression inside the brackets is 0 when it is computed to be negative; the TFV-DP formation rate must be strictly positive or 0). The second component is the rate of elimination or the conversion from TFV-DP to TFV governed by the rate constant *k*_off_.

Boundary and initial conditions for the system of Eqs. (9a)–(9e) are given in Eq. (10).

10a $$ {C}_G={\varPhi}_G{C}_E\ @\ \left(y=0,0\le x\ge L\right) $$

10b $$ \frac{\partial {C}_E}{\partial y}=0\ @\left(y=0,x>L\right) $$

10c $$ {C}_E={\varPhi}_E{C}_S,\ {D}_E\frac{\partial {C}_E}{\partial y}={D}_S\frac{\partial {C}_S\ }{\partial y}@\ \left(y={h}_E\right) $$

10d $$ \frac{\partial {C}_S}{\partial y}=0\ @\ \left(y={h}_E+{h}_s\right) $$

10e $$ \frac{\partial {C}_E}{\partial x}=0\kern0.5em @\kern0.5em \left(x=0\ \mathrm{and}\ x=d\right) $$

10f $$ \frac{\partial {C}_S}{\partial x}=0\kern0.5em @\kern0.5em \left(x=0\ \mathrm{and}\ x=d\right) $$

10g $$ {C}_G\left({t}_0\right)={C}_0 $$

10h $$ {C}_E\left({t}_0\right)=0 $$

10i $$ {C}_S\left({t}_0\right)=0 $$

10j $$ {C}_B\left({t}_0\right)=0 $$

10k $$ {C}_{DP}\left({t}_0\right)=0 $$

The coordinate system here is different from that in gel spreading computations; the origin is at the top left corner of the tissue layer. The gel is inserted at time *t*_*o*_, which is taken as 0 in the computations here. Partition coefficients between gel and epithelium and epithelial and stromal compartments are *Φ*_*G*_ and *Φ*_*E*_, respectively. Thicknesses of the epithelium and stroma are denoted *h*_*E*_ and *h*_*S*_, and *d* is the distance along the canal from introitus to fornix.

### Numerical solution of the equations of the model

We solve the system described in Eqs. (9) and (10) using Matlab [34]. Epithelial and stromal compartments are represented as rectangular regions with *xy* grids. After establishing the coordinates, we convert Eqs. (9a)–(9c) from a system of partial differential equations to a system of ordinary differential equations using centered difference formulas for first and second derivatives. We input results from the model for gel spreading and leakage described above. We determine the half-length *L* of the gel (Eq. 9a) in contact with the tissue surface from the gel spreading model. This length is discretized to fit into the space of the two-dimensional drug transport problem. The solution at each point in the space is now described by a system of ordinary differential equations that are solved in MATLAB using the Runge–Kutta 4,5 method. Output of the MATLAB computation gives concentrations of drug in gel, epithelium, and stroma in time and space. We solve Eq. (9d) separately from the rest of the system to find the drug concentration in the blood. The equation is a simple first-order ordinary differential equation with an input term that is dependent on the calculated stromal drug concentration. The solution to the TFV-DP term in Eq. (9e) is coupled with the solution to the tenofovir Eq. (9a–c) and is solved simultaneously in the overall system of differential equations.

### Parameters in the model

There are a number of parameters in this model (Table 3). These are largely the same parameters used in our initial analysis of the problem (with uniform coating along the entire length of the vaginal canal [14]). We take the epithelial and stromal layer thicknesses as 200 μm and 2.8 mm, respectively [35]. The model contains values of the length *d* and width *w* of the vaginal canal. These vary amongst women [30]. In applying the model, based on the range of human vaginal morphometric data, we consider three representative cases: an “average size,” a “small size,” and a “large size.” We use the small and large sizes to contrast rates and extent of coating by the three test gels. We use the average size in performing follow-up mass transport computations of drug concentration distributions. These latter computations require values for the three rate constants: for gel dilution, drug loss to the vasculature in the stroma, and drug loss from the bloodstream, *k*_*D*_, *k*_*B*_, and *k*_*L*_, respectively. We obtained these using comparisons of model predictions for the clinical tenofovir gel with human pharmacokinetic (PK) data for drug concentrations in vaginal tissue biopsies and blood samples [27]. We apply those same values here. Using Stokes Einstein theory, we deduced values of the diffusion coefficient to be 6 × 10^−6^ cm^2^/s in the gels. All are highly hydrated, and the relatively small differences in diffusion coefficient that might occur across the three gels have a minor impact on our drug transport computations (simulation data not shown). We used the values of diffusion coefficients for tenofovir in epithelium and stroma as 7 × 10^−8^ and 4 × 10^−7^ cm^2^/s, respectively, and the value of 0.75 for the partition coefficient at the interface between gel and epithelium. These were derived from initial experimental measurements of tenofovir transport in specimens of fresh porcine vaginal tissue using confocal Raman spectroscopy [36]. The kinetic parameters for TFV-DP, *k*_on_, and *k*_off_ were estimated based on PK studies of TFV-DP formation [37]. We note that these studies measured varying TFV-DP clearance rates in different cell types; however, all are quite slow (TFV-DP is retained over the order of days). Such differences in the values of *k*_off_ for TFV-DP have negligible effect on results here, which are limited to 24 h after gel application. The value of *V*_*B*_, volume of distribution in the blood compartment, was taken as 75 L [33].

**Table 3:** Parameters used in the drug mass transport model

Parameter	Symbol	Value
Gel diffusion coefficient (cm^2^/s)	*D*_*G*_	6 × 10^−6^
Diffusion coefficient for TFV in epithelium (cm^2^/s)	*D*_*E*_	7 × 10^−8^
Diffusion coefficient for TFV in stroma (cm^2^/s)	*D*_*S*_	4 × 10^−7^
Gel/epithelium partition coefficient	*Φ*_GE_	0.75
Epithelial thickness (cm)	*h*_*E*_	0.02
Stromal thickness (cm)	*h*_*S*_	0.28
Rate constant due to dilution in gel (h^−1^)	*k*_*D*_	1.22
Rate constant to blood vessels in stroma (h−^1^)	*k*_*B*_	0.119
Rate constant for loss in blood (h−^1^)	*k*_*L*_	1.41
Rate of formation of TFV-DP (h−^1^)	*k*_on_	0.693
Rate of elimination of TFV-DP (h−^1^)	*k*_off_	0.00413
Volume fraction of host cells in epithelium, stroma	*φ*_*e*_, *φ*_*s*_	0.95, 0.1
Proportion of TFV-DP converted from TFV	*r*	0.1
Initial concentration in gel (ng/ml)	*C*_*0*_	10^7^
Volume of distribution for blood (L)	*V*_*B*_	75
Length of vaginal canal for small, average and large vagina (cm)	*d*	12, 13, 15
Width of vaginal canal for small, average and large vaginal (cm)	*w*	3, 3.35, 3.5
Surface area for small, average, large vagina (cm^2^)	*A*	72, 87.1, 105

## Results

### Gel flow along the lumen of the vaginal canal

**Fig. 3:**
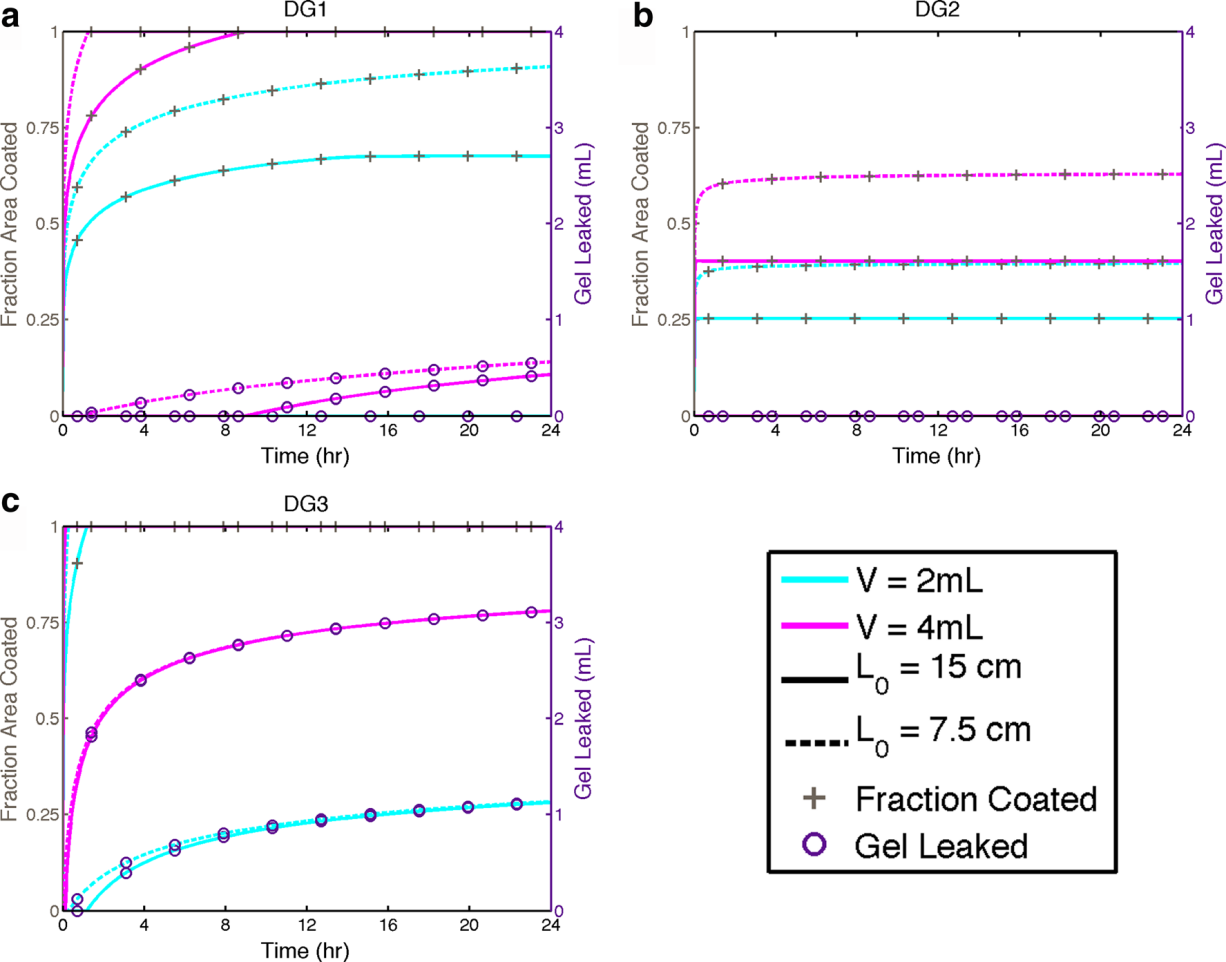
Fractional area coated and leakage volume over time for gels DG1(**a**), DG2(**b**), and DG3(**c**) inserted at position L_0_ in the canal with volumes 2 and 4 mL. These results are for a morphometrically large vaginal canal, length *d* = 15 cm and width *w* = 3.5 cm. L_0_ = 15 cm corresponds to gel insertion into the fornix, and L_0_ = 7.5 corresponds to gel insertion to the midpoint along the vaginal canal. Gels DG1 (2 mL) and DG2 (both volumes) do not leak. Gel DG3 spreads very rapidly, and both volumes achieve complete coating within 72 min

**Fig. 4:**
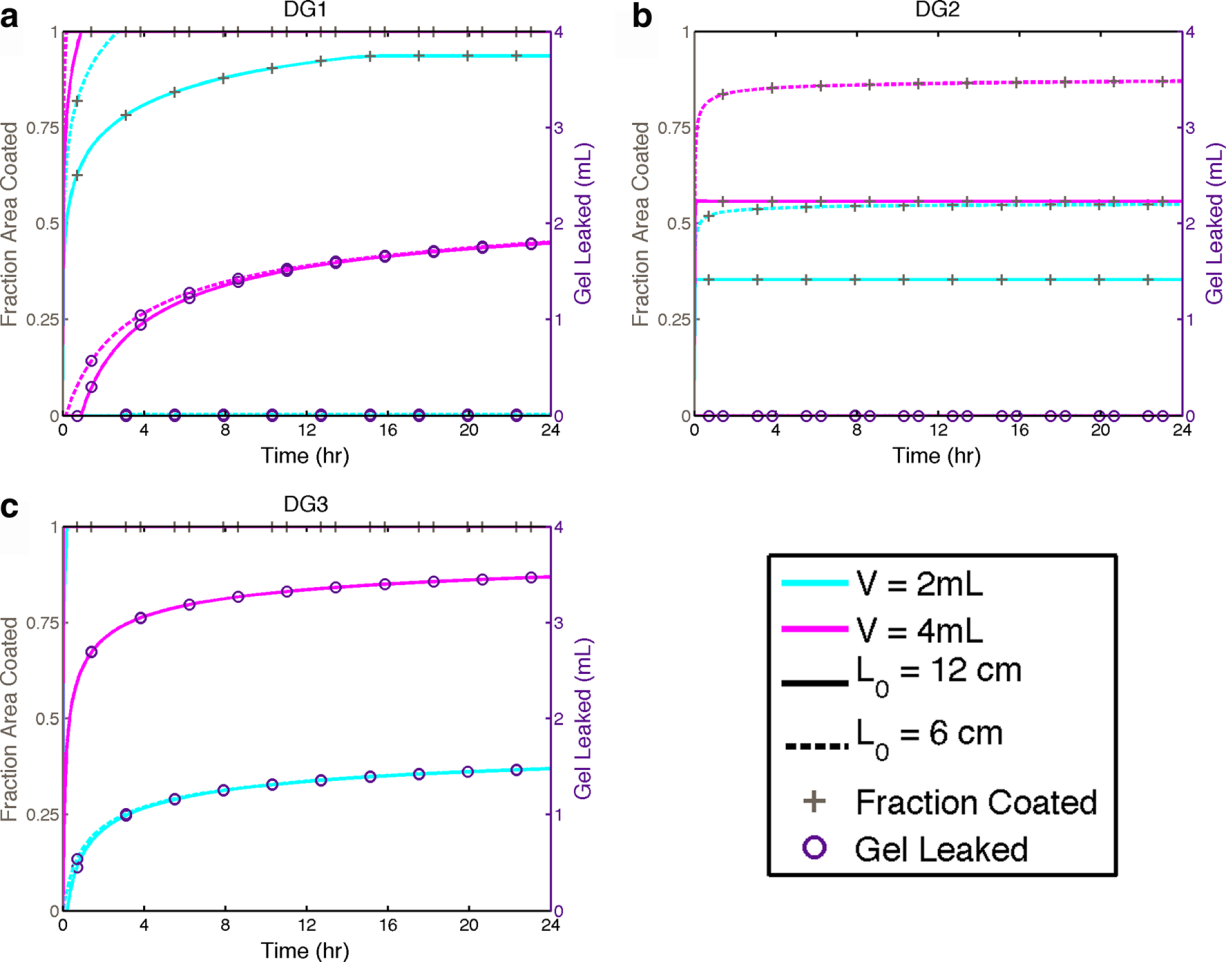
Fractional area coated and leakage volume over time for gels DG1(**a**), DG2(**b**), and DG3(**c**) inserted at position L_0_ in the canal with volumes 2 and 4 mL. These results are for a morphometrically small vaginal canal, length *d* = 12 cm and width *w* = 3 cm. L_0_ = 12 cm corresponds to gel insertion into the fornix, and L_0_ = 6 corresponds to gel insertion to the midpoint along the vaginal canal. Gels DG1 (2 mL) and DG2 (both volumes) do not leak. Gel DG3 spreads very rapidly, and both volumes achieve complete coating within 15 min

Flow of a gel bolus over the epithelial surfaces of the vaginal canal can be characterized by the instantaneous length of the bolus along the surfaces as a function of time; this is proportional to the coated surface area in our rectilinear model. Two salient measures of the flow are the time-dependent percent of the total epithelial surface coated by a gel and the gel volume that has leaked out from the introitus. Figures 3 and 4 plot these two measures for the three test gels DG1, DG2, and DG3 for volumes 2 and 4 mL. Those volumes span the range of potential vaginal microbicide gel volumes being considered. The two different vaginal sizes are termed “small” (length *d* = 12 cm, width *w* = 3 cm, surface area *A* = 72 cm^2^) and “large” (length *d* = 15 cm, width *w* = 3.5 cm, surface area *A* = 105 cm^2^). Two different sites of initial placement of the gel bolus are considered: one at the inner end of the canal at the fornix (l_0_ = 12 cm and l_0_ = 15 cm) and one midway along the canal (l_0_ = 6 cm and l_0_ = 7.5 cm) for small and large sized canals, respectively. Results are plotted up to 24 h after gel insertion in order to display the approach to steady state of gel spreading. As seen in the shapes of the plots, coated area achieves a nearly steady-state value or gel begins to leak by about 12 h. Further, we caution that these computations model gel spreading in the absence of significant body motions by the user and thus likely apply to intervals no greater than 8–12 h (this limitation in gel flow analysis has minimal effect, however, on drug transport within the mucosal layers at times up to 24 h; see “Discussion).”

Gel DG3 is the least viscous and exhibits no yield stress; thus, this gel spreads the fastest. DG2 is the opposite, having a high yield stress and exhibiting a higher viscosity over the range of shear rates expected in vivo. This gel was created with the intention that it flows very slowly and thus remains near the site of insertion. Thus, although it does not tend to spread, this gel is also much less likely to leak. The rheological properties of gel DG1 are intermediate between those of DG2 and DG3. Although this gel does not have a significant yield stress, it does not spread as rapidly as DG3. The spreading kinetics of all gels are similar in that there are a short time scale during which spreading is relatively rapid and a longer time scale over which the spreading rate is much slower. Depending upon the site of placement, gel volume, and rheological properties, the gel may begin to leak during flow over either of these two time scales. If a yield stress is present, the spreading rate eventually diminishes to zero, at which the squeezing force and the gel resistance to deformation embodied by the yield stress are in equilibrium.

There are differential interactions amongst gel properties, volume, vaginal size, and site of gel insertion. The spreading and leakage plots for DG1 in (Figs. 3a and 4a) show that at 4 mL, this gel spreads relatively rapidly, completely coating the lumen in 2–8 h after insertion, depending upon site of insertion and vaginal size. However, it also exhibits leakage of about 0.5–1 mL over 24 h depending again upon site of insertion and vaginal size. Applying a 2 volume of gel DG1 results in lower coated area which is more dependent on initial position. The 2-mL volume produces no leakage over 24 h.

Given its relatively high yield stress, gel DG2 does not coat completely over 24 h and thus does not leak (Figs. 3b and 4b). The coated area is clearly dependent on volume and site of placement. By 24 h, a 2-mL volume coats between about 35 and 60 % of the epithelial surface and 4 mL coats between 45 and 75 %, depending upon the site of placement and vaginal size.

Figures 3c and 4c show clearly that gel DG3 flows very rapidly. Both volumes of this gel coat the entire epithelial surface within 72 min, and it also leaks to a very high extent. By 24 h, only about 0.8 mL of the initial volumes of this gel is retained within the vaginal canal, and the initial position has little effect, especially at longer times.

### Drug transport into the epithelial and stromal layers of the tissue

#### Multidimensional, time-dependent tenofovir and tenofovir diphosphate concentration distributions in epithelium and stroma

**Fig. 5:**
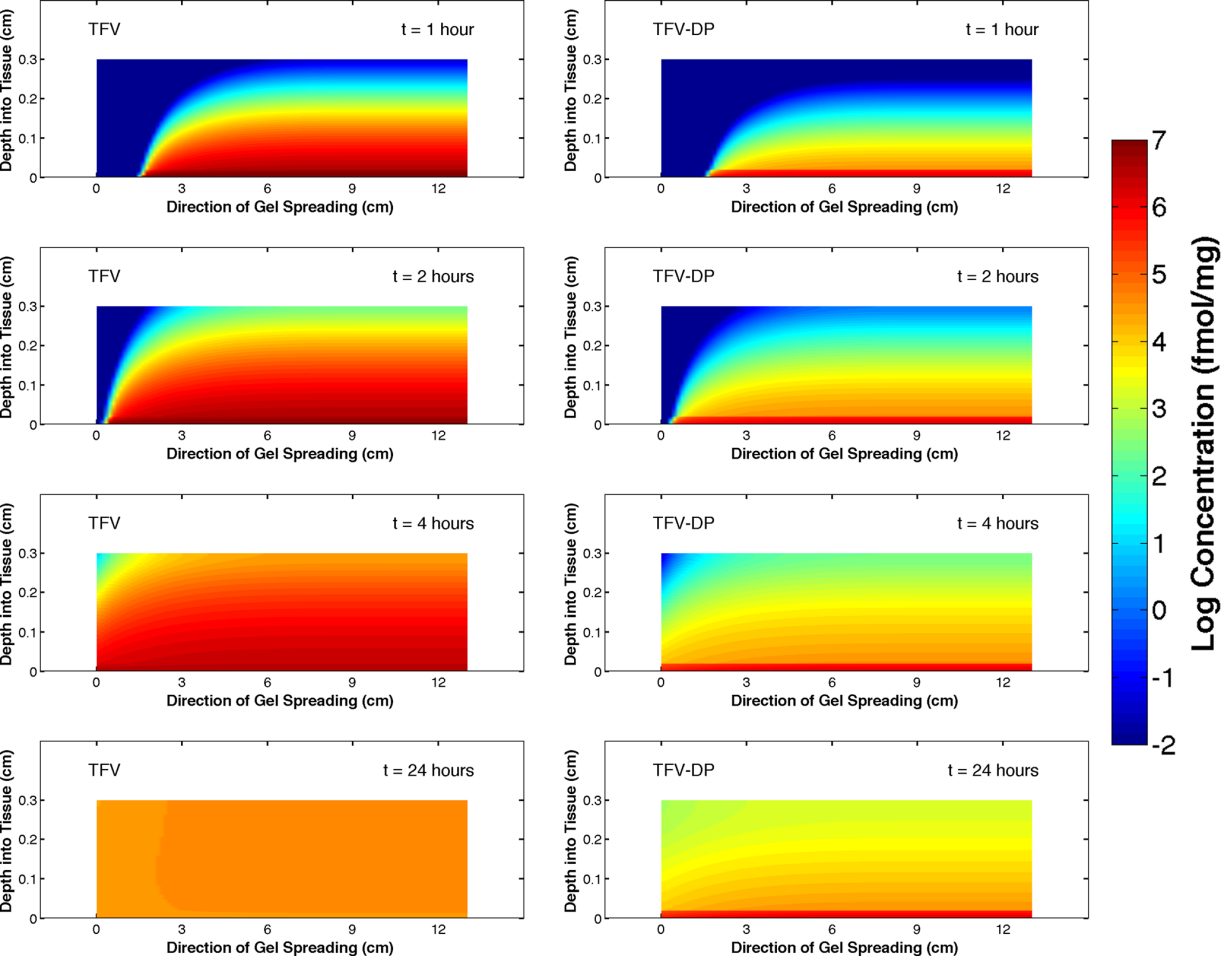
Concentration distributions in tissue at 1, 2, 4, and 24 h for a 4-mL DG1 gel inserted at the inner end of a vaginal canal with length and width, 13 and 3.35 cm, respectively (morphometrically average dimensions, see text). The epithelium is 0.02-cm thick and the stroma is 0.28-cm thick. The gel spreads from right to left. The *colors* represent local drug concentration on the log scale plotted over the range <10^−2^ to 10^7^ fmol/mg (see attached video for full 24 h animation)

Drug transport from a spreading gel into the mucosal tissue can be characterized by a spatio-temporal map of concentration, here in two dimensions (depth into tissue and distance along the vaginal canal) and time. This is illustrated by an animation for 3 mL of gel DG1 (inserted at the inner end of a vaginal canal of average dimensions; see Supplemental Material). Still, images at 1, 2, 4, and 24 h are shown as heat plots in Fig. 5. Here, thicknesses of the epithelial and stromal layers are 0.02 and 0.28 cm, respectively. The *x*- and *y*-axes are in directions longitudinal and transverse to the tissue surface; *x* ranges from 0 cm at the introitus to 13 cm at the innermost end of the canal, and tissue depth ranges from 0 cm at the tissue surface to 0.3 cm at the lower margin of the stroma.

Concentration distribution of TFV longitudinally in tissue closely maps with the longitudinal extent of gel spreading. The leading edge of gel (Fig. 5) is effectively the leading edge of the TFV concentration profiles. Peak concentrations in tissue diminish over time, as gel spreads further along the canal and as drug diffuses down into the tissue and is cleared within the stromal layer. At times up to 4 h, the maximum concentration in the tissue is about 10^7^ fmol/mg, and concentration remains at above 10^5^ fmol/mg throughout most of the stroma. At 24 h, the tissue has a concentration between 10^4^ and 10^5^ fmol/mg. The longitudinal concentration distribution of TFV-DP also maps with gel spreading. Concentration (per unit tissue volume) is higher in the epithelium than stroma because of the higher concentration of cells. Overall, TFV-DP concentration distribution follows that of TFV for relatively short times. However, for longer times (viz > 8 h), TFV-DP concentration approaches a steady-state value because of its very low clearance rate.

#### Volume-averaged concentrations in compartments vs. time

**Fig. 6:**
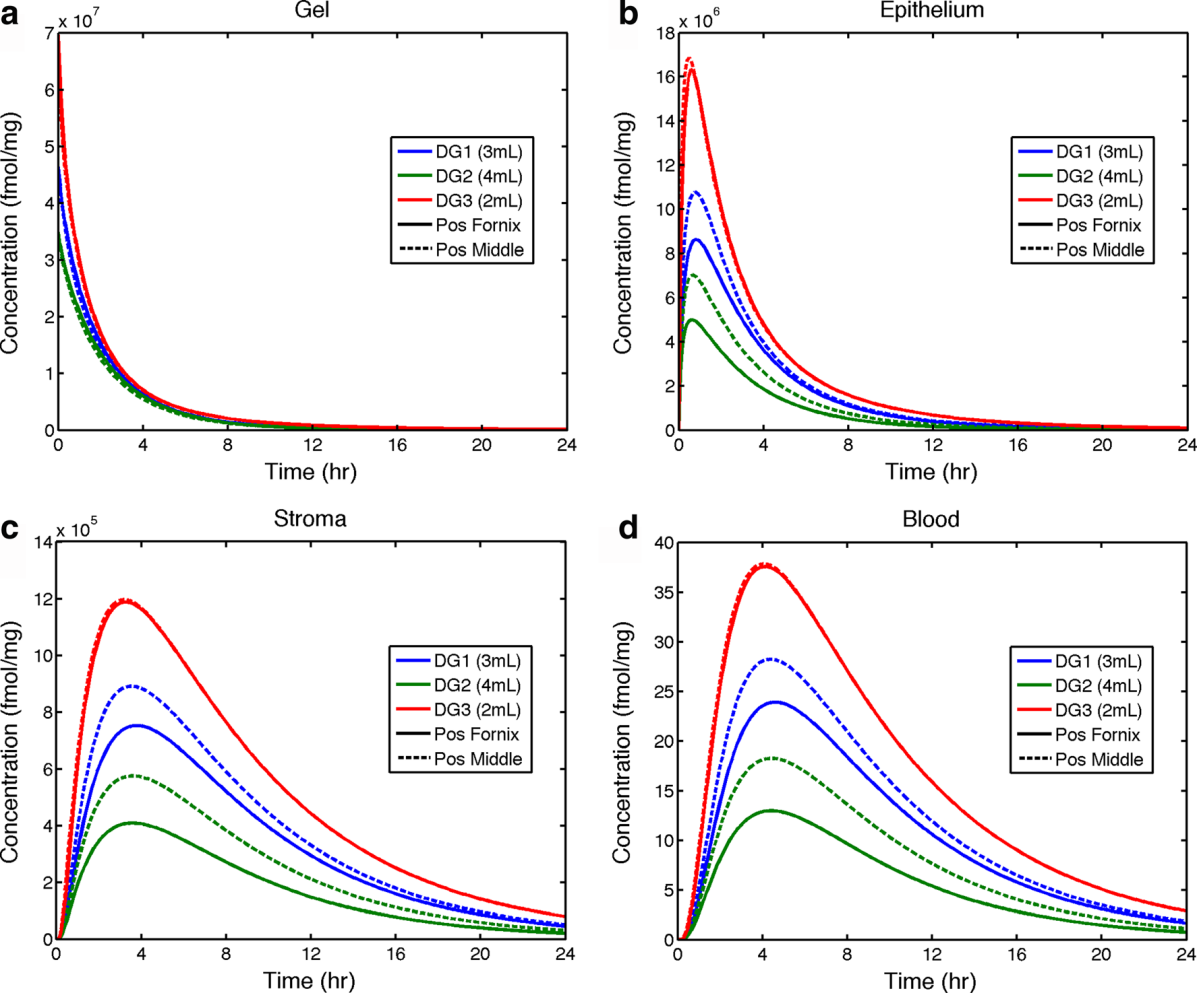
Volume-averaged TFV concentrations in gel, epithelium, stroma, and blood compartments over time for the three gels inserted to the middle and innermost end (the fornix) of a vaginal canal with average morphometric dimensions (see text). Total mass of loaded drug is conserved over all gel volumes, M_o_ = 40 mg

**Fig. 7:**
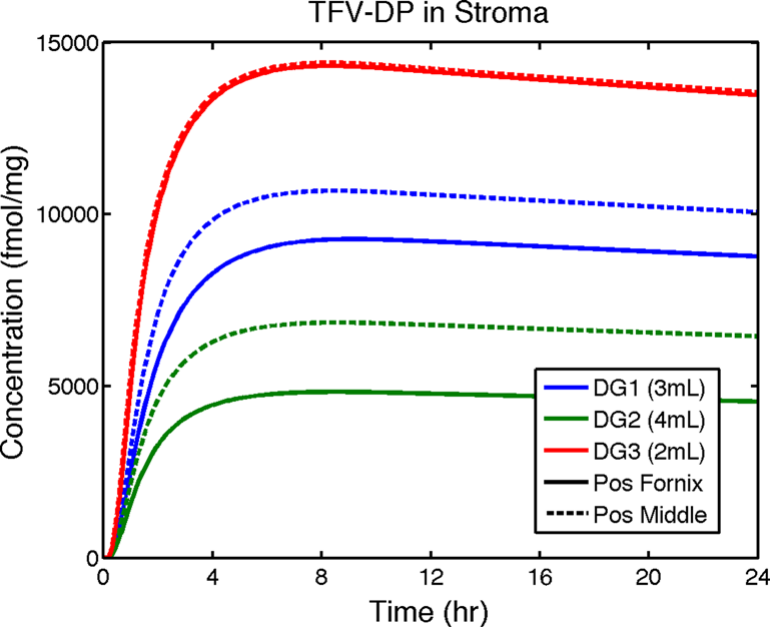
Volume-averaged concentrations of TFV-DP in stroma over time for the three gels inserted to the middle and innermost end (the fornix) of a vaginal canal with average morphometric dimensions. Total mass of loaded drug is conserved over all gel volumes, M_o_ = 40 mg

Volume averages vs. time of drug concentration distributions in each compartment (gel, epithelium, stroma, and blood [in which concentration is homogeneous]) yield plots analogous to measurements of drug concentrations in experimental pharmacokinetic studies [27]. Figures 6 and 7 give examples of such plots for gel volumes 3 mL (DG1), 4 mL (DG2), and 2 mL (DG3). We chose volumes for each gel (within the range 2–4 mL) that give coating at 24 h which is closest to the surface area of the vaginal canal. While not strict optima, the three selected values of gel volume do provide an instructive example of conditions that illustrate pharmacologically relevant outputs of the model. In these computations, we used dimensions representing an averaged sized vagina: length = 13 cm and width = 3.35 cm; epithelial thickness was again 0.02 cm. Here, we conserved total mass of loaded drug in gel across volumes (an alternative example would have been to conserve initial concentration; see additional computations below). Thus, initial drug concentration is inversely proportional to gel volume. We chose a value M_o_ = 40 mg, which is the tenofovir mass in the 4-mL volume of the clinical microbicide gel used in trials [8, 38].

From Fig. 6, we see that insertion of all gels to the fornix gives lower TFV concentrations in the epithelium and stroma than insertion to the middle of the canal. Stromal AUC values are about 15, 30, and 1 % lower for gels DG1, DG2, and DG3 (computations not shown). From Fig. 7, we see that average stromal concentrations of TFV-DP follow the same pattern for all three gels. They rise to a quasi-steady-state value in about 6 h for all gels and then remain nearly constant beyond the interval of computation (because of the very low loss rate constant for TFV-DP). Peak values of TFV-DP concentrations are higher for gel insertion to the middle of the canal by about 15, 40, and 0.5 % for gels DG1, DG2, and DG3, respectively. Thus, results for gel DG3 (the lowest viscosity, relatively “runny” gel) are relatively insensitive to site of insertion because it coats the entire length of the canal so rapidly. Overall, results in Fig. 7 indicate that the rank ordering of effective drug delivery performance (i.e., TFV-DP concentrations in stroma) for the gels is DG3 (2 mL) > DG1 (3 mL) > DG2 (4 mL).

#### Volume of stromal layer containing HIV prophylactic concentrations of tenofovir diphosphate

**Fig. 8:**
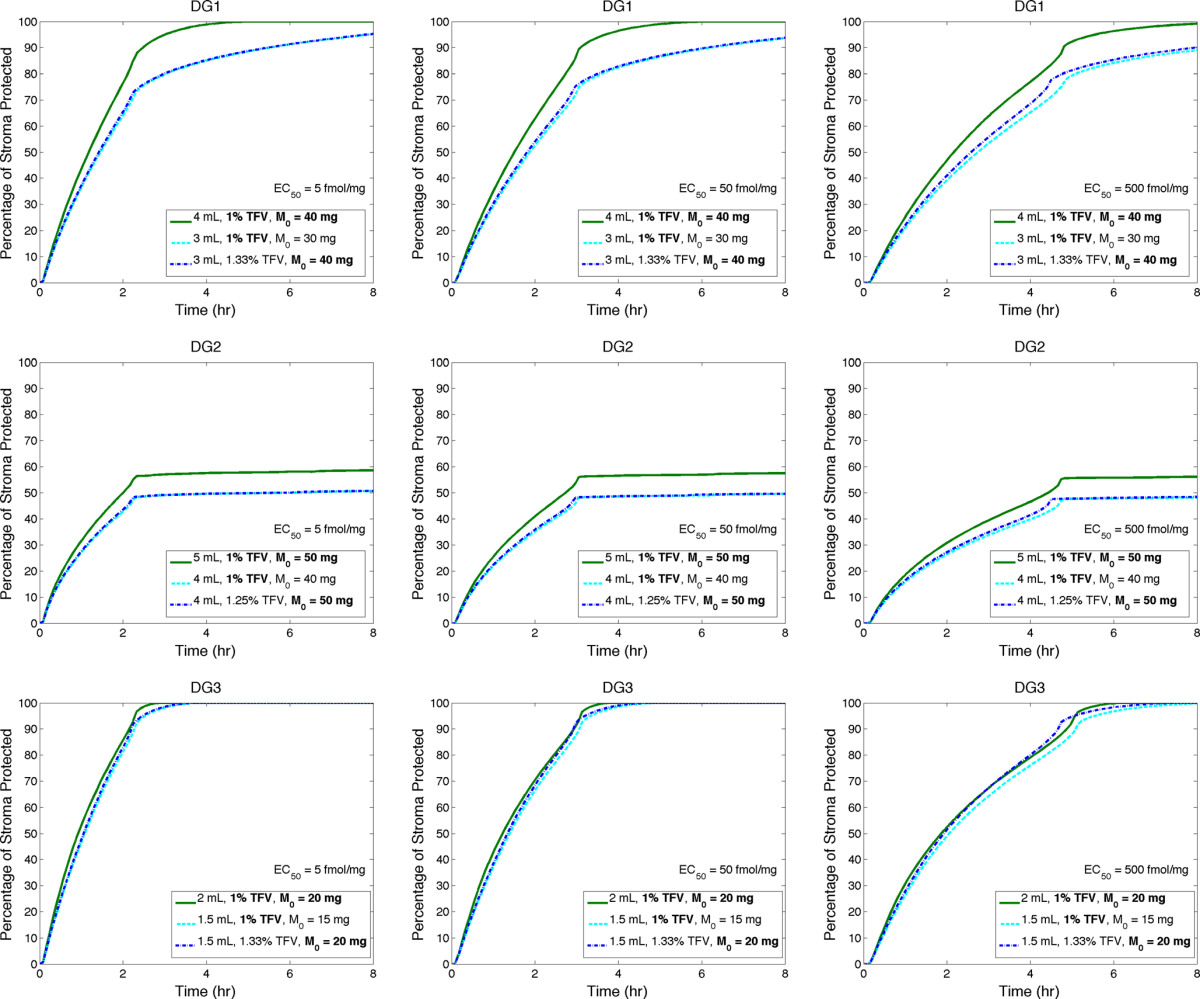
Plots of percent protected vs. time for the three test gels, DG1, DG2, and DG3. For each gel (*different rows*), the reference EC50 for tenofovir diphosphate is varied by 2 logs (*different columns*) as 5, 50, and 500 fmol/mg. Further, three combinations of gel volume and initial tenofovir concentration (C_o_) are considered: (*1*) a reference combination (*green line*) intended to produce the highest percent protected per gel, (*2*) conservation of C_o_ from condition 1 and reduction of gel volume (so that M_o_, the mass of loaded TFV, is conserved), and (*3*) conservation of M_o_ from condition 1 (so that initial concentration C_o_ is increased)

The pharmacodynamic potency of tenofovir is manifest through concentrations of tenofovir diphosphate in HIV-infectible cells. Many diverse studies have investigated this; the great majority of which focused upon the relationship between tenofovir concentrations in infectible tissue and the infectibility of that tissue. A one log range of EC50 values for tenofovir has emerged [33]. Fewer studies have focused upon the relationship between concentrations of tenofovir diphosphate and HIV infectibility. The problem is compounded by the fact that different HIV-infectible cell types produce different intracellular TFV-DP concentrations, that such production in cells in the female reproductive tract is regulated by estradiol and/or progesterone, and that different cell types appear to have different inherent TFV-DP dose response behavior. In the analysis here, we focus on a 2 log range of the putative EC50 value for TFV-DP in stromal host cells. We compute the fraction of the total volume of the stromal layer (and, thus, the host cells therein) that instantaneously achieves a TFV-DP concentration ≥ this EC50 reference value. This fraction, termed percent protected, may thus relate to prophylactic effectiveness against HIV. It is an objective, mechanistically derived measure that can be related to the characteristics of the gel, drug, vaginal environment, etc. (viz. as presented above for measures of gel spreading, tenofovir transport, and tenofovir diphosphate production). We based the range of EC50 values for tenofovir diphosphate by referencing the lower bound value of 0.5 μM for tenofovir [33] TFV-DP concentrations in human vaginal biopsies after dosing with the 1 % TFV gel are approximately two logs lower than the measured TFV concentrations. Hence, we chose 0.005 μM (5 fmol/mg) as the lower bound of the EC50 for TFV-DP here. We conservatively used an upper bound for this EC50 value for TFV-DP that is 2 logs higher. Figure 8 illustrates application of this computation to gels DG1, DG2, and DG3, varying gel volume and tenofovir loading. This exercise provides insight into practical decisions that developers must make, once a gel composition has been decided, about what volume to apply and whether variations in loaded drug concentration (compatible with safety, stability, and solubility requirements) are needed. For each gel, we used, as a reference condition, a volume giving rise to a coated area in 1 h as close as possible to that of the vaginal canal; for this reference condition, loaded TFV concentration was 1 % (as in the clinical TFV gel). Because gel DG2 coats so slowly, we did increase its reference volume from 4 to 5 mL (vs. results shown in Figs. 6 and 7). We then perturbed either the reference gel volume or TFV concentration: Volume was reduced while maintaining TFV concentration (termed “TFV concentration conserved”) or volume was reduced, but concentration was altered to achieve the same mass of drug loaded into gel as for the reference condition (termed “TFV mass conserved”). Average vaginal dimensions of 13 by 3.35 cm were used to represent an average size, and gel was inserted into the fornix.

From Fig. 8, we see a steep rise in percent protected during the first 2–4 h after gel insertion; after which, the curve bends over and approaches a maximum value over a range of times from almost instantaneous (gel DG2) to several hours (gel DG1). Thus, protection evolves initially at a fast rate during an interval in which at least 90 % of its maximum value of percent protected is reached. This peak value is sustained at near steady state because of the very slow clearance of TFV-DP from cells. The time scale of analysis here is ≤24 h post-gel application. Although there appears to be some variation in the duration of clearance rate of TFV-DP amongst cell types, all reported rates are on time scales much greater than 24 h [39, 40]. Consequently, differences amongst them have minimal impact on the curves in Fig. 8. We can characterize the behavior of percent protected in terms of the time to reach the bend-over point in the curve (an effective t_bend_) and the peak value of percent protected (an effective PP_max_ at the longer, quasi-steady-state times). For all gels, the highest gel volume achieves the highest percent protected the most rapidly. Reducing that volume (e.g., for cosmetic reasons) while conserving either loaded TFV concentration or mass of TFV loaded produces indistinguishable results. That is, gel volume, not loaded TFV concentration, is the dominant factor. However, quantitative differences across volumes and drug loadings are negligible for gel DG3. This gel, which has a low viscosity, achieves peak values (100 %) the fastest across the three gels, even though the mass of drug in it is the lowest across the conditions considered. This is because the gel spreads so rapidly, creating a thin coating layer across which TFV diffuses more rapidly than in the thicker layers for the other two gels. The higher volume (4 mL) for gel DG1, loaded at 1 % TFV, achieves ~100% protection, but more slowly than for DG3. Reducing its volume reduces the peak value to about 90 %. This results from the competition between increased TFV delivery due to increased coating and the loss of TFV due to gel leakage plus clearance of TFV in the stromal layer. Gel DG2, the very high viscosity gel, never achieves more than 60 % protected because it only coats a fraction of the length of the vaginal canal. For all gels, the effect of increasing the threshold EC50 value for protection is to increase the time interval over which the steep rise in percent protected occurs (t_bend_ increases); however, this 2 log variation of the EC50 has little effect on the maximum value achieved of percent protected (PP_max_).

## Discussion

Topically acting microbicides are an important modality that could complement vaccines in the fight to stem the AIDS pandemic. Gels have been a primary delivery platform being developed for vaginal microbicide application. However, no gel, to date, has successfully completed phase 3 trials. Failures of adequate participant adherence in the VOICE and FACTS trials raise the question of whether or not the gel dosage form can achieve adequate willingness to use in target populations. Thus, it is logical to consider what characteristics of gels, their design, and their application processes might be improved to foster better willingness to use without compromising prophylactic activity. The deterministic approach embodied in the modeling here—which computes and links gel spreading/leakage to drug delivery—could contribute to evaluation of the prospects for improved design of gels and their applied volumes. Improved gels could, for example, be less messy and more discreet, while still achieving effective PK and PD. By helping in prediction of the interval of maximum protection after gel insertion, the model here could also inform instructions to users about proper gel use. Although the computations were for tenofovir, the deterministic approach here is robust to multiple drugs. The computational model here is also for conditions in which gel is applied prior to sexual activity. Interpretation of the percent protected measure here can nonetheless address salient questions about gel use to prevent vaginal HIV transmission, viz: (1) how long the users must wait following gel insertion and prior to sex to achieve protection (captured in the t_bend_) and (2) how good that protection is (captured in the PP_max_).

We note that computations here extend to 24 h post gel insertion. Towards the end of this interval, details of gel spreading per se likely become more complex than as simulated in our model, as a woman’s activity contributes additional forces against the gel, and further dilution with ambient fluids occurs. However, we also note that drug remaining in a gel at those later times is nearly depleted (Fig. 6) and lumenal drug in the vagina has minimal impact on drug transport through the mucosal layers at such times. Consequently, we believe that our computations of drug distribution in epithelium and stroma (viz. Figs. 6 and 7) are not compromised.

### Gel spreading

Results in Figs. 3 and 4 show broad ranges in rate and extent of gel spreading and leakage across the three gels, three volumes, and two sites of initial gel placement. There are currently no standards for rheological properties or volumes of vaginal (or rectal) microbicide gels, and it was thus logical to create these test gels to embody a broad range of spreading behavior. The rate of spreading has an inverse relationship with gel viscosity and yield stress and a direct relationship with gel volume, over variations in vaginal canal size and site of gel placement (Figs. 3 and 4). Of course, vaginal canals of greater length and width will accommodate larger gel volumes without leakage.

### Drug mass transport

Mass transport of tenofovir from a gel is a time- and space-dependent process, within as well as between compartments (Fig. 5). Because drug mass transport longitudinally along the canal maps with the position of the leading edge of the gel, relatively good spreading (gels DG1 and DG3) ensures drug distribution along the length of the vaginal mucosa while limited gel spreading (gel DG2) delays such distribution, the presence of vaginal fluid notwithstanding.

Because total mass of TFV and TFV-DP in epithelium and stroma scales with rate of gel spreading, the curves for average TFV and TFV-DP concentrations have the same qualitative shape across gels (Figs. 6 and 7). The curves for TFV look very different from those for TFV-DP because the long half-life of TFV-DP in cells causes its average concentration in stroma to plateau at about 4–6 h. In contrast, average TFV concentrations in epithelium, stroma, and blood rise to C_max_ values and then decline, as supply from gel diminishes and drug is cleared in stroma and blood.

TFV and TFV-DP concentrations in epithelium and especially stroma exhibit sharp spatial concentration gradients with depth at early times (Fig. 5). Then, TFV concentration in the epithelium flattens out as drug in the gel is depleted during the first 2–3 h (cf. Figs. 5 and 6; see also Fig. 2 from reference 22). TFV in the stromal layer accumulates in the upper few hundred micrometers of thickness, and concentration distribution flattens out as it drops by about 2 logs during 24 h (Fig. 5; see also Fig. 2 from reference 22). As a result, TFV-DP concentration in the stroma is much higher in these upper few hundred micrometers of that layer than below; consequently, the spatial average concentration of TFV-DP throughout the depth of the stroma is relatively high (compared to the target EC50 values) regardless of the rate of gel spreading. This is discussed further in the next section.

We compared predictions using this model with human PK data for the 1 % tenofovir gel inserted at 4 mL for a vaginal of average dimensions [27]. Using our rheological data for that gel, we computed TFV concentrations vs. time in gel, epithelium, stroma, and blood compartments for up to 24 h post-gel insertion. Concentrations in epithelium and stroma were combined to simulate volume-averaged concentrations in biopsies, as would be obtained in a PK study [14]. We computed values of C_max_ and C_24_ for biopsy and blood compartments for direct comparisons with the PK data (median values given in Table 3 of reference [27]). Relative differences are given in Table 4.

**Table 4:** Differences between predictions of model for the clinical 1 % tenofovir gel applied at the fornix in a 4-mL volume to a vagina of average dimensions vs. human PK data [27]

	C_max_ (%)	C_24_ (%)
Biopsy	−5.5	12.2
Blood	19.4	−2.4

The differences between theory and experiment in Table 4 are very small compared to the variability in the PK data. We posit that the agreement between theory and experiment here is sufficiently good to motivate further applications of the model.

### Protective effect of TFV-DP

Computations here used an EC50 as a target concentration for computations of a measure of potential for HIV prophylaxis (the percent protected). A more conservative approach would be to use an EC90 value. However, because we evaluated a 2 log range of the EC50 value, we believe that computations here demonstrate the potential value of this approach in helping understand and quantify the conditions of prophylaxis. In particular, we found that increasing the target prophylactic TFV-DP concentration slows the post-gel insertion rise to the maximum potential for protection, but does not appreciably alter that level over 24 h. As seen in Fig. 7, the spatial average concentration of tenofovir diphosphate in the stroma rises rapidly with respect to the minimum threshold EC50 value, surpassing this at a time small compared to the effective t_bend_ for percent protected (Fig. 8). This is because percent protected is limited by how rapidly and completely the gel coats the length of the canal. Regions of mucosa not coated by gel have very little drug in them for extended times (because longitudinal drug diffusion in the tissue is very slow). As noted above, there are relatively high TFV-DP concentrations in the upper stroma, but these drop off rapidly with depth below. Thus, the lower regions of the stroma have very low TFV-DP concentrations at earlier times and this, as well as the finite rate of gel spreading, drives down the net volume of stromal tissue with protective TFV-DP levels at those times. Intromission likely smoothes out gel distribution and may extend completeness of coating along the canal. However, subsequent semen deposition may rapidly expose tissue to virus to the extent that uncoated, bare regions of the epithelial surfaces remain. Thus, in those regions, drug has little opportunity to diffuse into/through tissue prior to arrival of the virions. In contrast, in coated regions, the relatively slow HIV migration through the epithelium and past the upper few hundred mirometers of the stroma allows more time for protective TFV-DP to diffuse lower into the stroma. Overall, the percent protected measure captures the preponderance of how the time-space history of TFV-DP may neutralize HIV infection of stromal host cells, but not its entirety. Mathematically speaking, a bivariate characterization of both TFV-DP concentration distribution and the degree of gel coating might provide a more complete measure of protection.

### Summary

A general compartmental PK model was developed and applied to human vaginal delivery of tenofovir by a gel. The approach is fundamentally applicable to the range of microbicide drugs being evaluated. Multiple factors interact in non-linear ways to govern tenofovir delivery, production of tenofovir diphosphate, and the protective effect of the tenofovir diphosphate. The model here defines and integrates them in a deterministic way.

Results show that predictions of gel spreading per se inform us significantly about tenofovir delivery and consequent protection by tenofovir diphosphate, as well as about propensity for gel leakage. The new measure of protection, termed percent protected, provides for the first time an objective, mechanism-based metric that can be computed to assist in product design and evaluation. The time dependence of percent protected provides insights about the time, post-gel insertion, required to achieve maximal protection, the degree of that protection, and its duration (akin to classical pharmacological analysis of drug concentrations). Analysis using the percent protected metric here is striking in that it suggests that the low viscosity, runny gel would—despite it propensity for leakage and even with a smaller loaded drug mass—deliver more tenofovir initially and would as rapidly create prophylactic concentration distributions of tenofovir diphosphate as would a “typical” microbicide gel of intermediate viscosity (that even packages more drug). This is because thinner gel coating layers engender faster diffusion of drug into the epithelium than thicker ones. The implication is, for example, that a small volume of a microbicide gel that spreads well to effectively coat the vaginal canal and for which retention is promoted by properties such as bioadhesiveness (not included here, but addressable in this type of modeling) to inhibit leakage might be an alternative to the high volume microbicide gels previously evaluated in clinical trials. Overall, the new modeling approach here extends earlier efforts to create computational tools that can be used in the objective prospective design of new, improved microbicide products and in the retrospective design of existing ones.

## Electronic supplementary material

ESM 1 (PDF 195 kb)

ESM 2 (MPG 1318 kb)
