# Supplementary material for: Vaginal deployment and tenofovir delivery by microbicide gels
Source: Drug Deliv Transl Res. 2015 Apr 15;5(3):279–94. doi: 10.1007/s13346-015-0227-1 (PMC4420798; doi:10.1007/s13346-015-0227-1)
Supplement: Supplementary file 1 — (PDF 195 kb) [file 13346_2015_227_MOESM1_ESM.pdf]

Supplemental Material: Squeezing flow of a Carreau-like Fluid with a Yield Stress in the Vaginal Canal

Lubrication theory can be applied because channel and gel height are small compared to length[1, 2]. The primary force driving the flow is squeezing due to the elasticity of the vaginal walls, which, for linear elasticity and the small deformations in lubrication theory, is proportional to the volume of gel applied[2]. Gravity has a relatively small effect and is neglected[2].

The following system of equations governing flow is obtained.

$$\frac{\partial v_x}{\partial x} + \frac{\partial v_y}{\partial y} = 0 \quad (1)$$

$$\frac{\partial P}{\partial x} = \frac{\partial}{\partial y}(-\tau) \quad (2a)$$

$$\frac{\partial P}{\partial y} = 0 \quad (2b)$$

Equation (1) is continuity, and equations (2a) and (2b) are the x and y momentum equations; v_x and v_y are velocities in the x and y directions, P is pressure, and τ is the xy shear stress. The squeezing force F acting on the upper and the lower surfaces of the gel is given by equation (3)

$$F = w\int_{-L}^{L} P dx \quad (3)$$

The value of F is a given quantity, as in our prior analyses of vaginal squeezing flows [1, 3]. Here w is the width (in the z direction) of the vaginal canal. The variable L is defined as the half-length of the gel; the integral is double sided with respect to the center of the gel.

The boundary conditions are given for the upper half plane of the flow (symmetrical about $y = 0$).

$$v_y = \dot{h} \ @ \ y = h \quad (4a)$$

$$v_x = 0 \ @ \ y = h \quad (4b)$$

$$\frac{\partial v_y}{\partial y} = 0 \ @ y = h \tag{4c}$$

$$v_y = 0 \ @ \ y = 0 \tag{4d}$$

$$P = 0 \ @ \ x = L \tag{4e}$$

Equations (4a) and (4b) give the no-slip boundary condition at the upper wall, where $\dot{h}$ is the velocity at which the wall moves as it squeezes against the gel (this is negative at $y = h$). Equation (4c) also follows from no slip, and equation (4d) follows from symmetry about the centerline of the flow ($y = 0$). As noted above, we assume that the pressure is atmospheric at the introitus and inner wall of the fornix in equation (4e).

For the constitutive relationship between shear stress and shear rate we use the Carreau-like model with a yield stress in equation (5), as implemented earlier by Tasoglu et al[4-6]. This model embodies rheological behavior typically associated with complex polymers used for vaginal gels, including a yield stress, shear thinning, and a finite zero shear viscosity. The Carreau-like model is preferred to the Carreau model because a constitutive relation of strain as a function of stress is more tractable for solving the equations than stress as a function of strain. The model contains 4 parameters: τ_0 , m_0 , m and n .

$$f(\tau) = \frac{\tau - \tau_0}{m_0} + \left(\frac{\tau - \tau_0}{m}\right)^{\frac{1}{n}} \ \tau \geq \tau_0 \tag{5a}$$

$$f(\tau) = 0 \ \tau < \tau_0 \tag{5b}$$

Equation (2b) implies the pressure is a function of *x* only, and from equation (2a) one can deduce the form of the shear stress as the product of y and the pressure gradient in the *x* direction.

Integrating continuity (1) using the boundary conditions (4a), (4b), and (4c), we obtain an integral equation relating the constitutive function f to x and the vertical wall velocity.

$$y_{crit} = \tau_0 / \tfrac{\partial P}{\partial x} \qquad (6a)$$

$$\int_{y_{crit}}^{h} \int_{y}^{h} f\left(\tfrac{\partial P}{\partial x}\tilde{y}\right) d\tilde{y}\, dy = -\dot{h}x \qquad (6b)$$

In equation (6a) y_{crit} is distance above the centerline at which the fluid has yielded. Equation (6b) is an integral equation for the pressure gradient. This is solved by combination with equations (3) and (6). After a series of integral and algebraic manipulations, we obtain the Reynolds equation for the problem:

$$\frac{1}{3}\frac{1}{m_0}\left(\frac{\partial P}{\partial x}h^3 - \tau_0 y_{crit}{}^2\right) - \frac{\tau_0}{m_0}h\left(h - y_{crit}\right) + \frac{\left(\left(\tfrac{\partial P}{\partial x}h - \tau_0\right)/m\right)^{\frac{2n+1}{n}}}{\left(\tfrac{\partial P}{\partial x}/m\right)^2\left(\left(2n+1\right)/n\right)} = -\dot{h}x \qquad (7)$$

The pressure gradient is usually given as a function of position x. However for equation (7) it is convenient to use an inverse function to give x as a function of pressure gradient. We define a new function g, that accomplishes this, Equations (8a) and (8b). The order of integration in equation (7b) is changed to take advantage of this. The double integral can also be simplified to a single integral, since the integrand is a function of one variable only. The result is given in equation (8c), where ξ_L is the pressure gradient at position $x = L$.

$$\frac{\partial P}{\partial x} \equiv \xi \qquad (8a)$$

$$g(\xi) \equiv f^{-1}(\xi) \equiv x \qquad (8b)$$

$$\frac{F}{2w} = \int_0^L \int_x^L f(\tilde{x})\, d\tilde{x}\, dx = \int_0^{\xi_L} \frac{1}{2}L^2 - \frac{1}{2}g(\xi)^2\, d\xi \qquad (8c)$$

Equation (8c) simplifies to an integral equation for the unknown wall velocity $\dot{h}$ (from equation (7)) as a function of time. Computationally, equation (8c) is solved by inputting a bounded guess to the solver “fzero” in Matlab[7]. Finally $\dot{h}$ is integrated at each time step to get h.

Our research group has developed, in parallel, vaginal gel flow theory that also considers a 2D channel geometry in an infinite spatial domain, with tissue walls as deformable, linearly elastic materials that exert a local pressure which is proportional to local wall distension in the *y*-direction[4, 2, 5, 6]. Here we have harmonized our 2 gel flow models by scaling the force in the parallel wall model to be proportional to gel volume, with constant of proportionality based on our elastic wall analysis; that is, the net force on a given gel volume is the same in the 2 models. We have compared the spreading behavior of the rectilinear and elastic wall models for the 3 test gels here (see next section). Results are relatively close; over 24 hrs of flow, instantaneous values of coated area differ by < 5% for gel DG1, and by ≤ 10% for gels DG2 and DG3.

References

1. Kieweg SL, Katz DF. Squeezing flows of vaginal gel formulations relevant to microbicide drug delivery. Journal of Biomechanical Engineering-Transactions of the Asme. 2006;128(4):540-53. doi:Doi 10.1115/1.2206198.
2. Szeri AJ, Park SC, Verguet S, Weiss A, Katz DF. A model of transluminal flow of an anti-HIV microbicide vehicle: Combined elastic squeezing and gravitational sliding. Phys Fluids. 2008;20(8):083101. doi:Artn 083101
Doi 10.1063/1.2973188.
3. Katz DF, Gao Y, Kang M. Using modeling to help understand vaginal microbicide functionality and create better products. Drug Delivery and Translational Research. 2011;1(3):256-76.

4. Tasoglu S, Park SC, Peters JJ, Katz DF, Szeri AJ. The consequences of yield stress on deployment of a non-Newtonian anti-HIV microbicide gel. J Non-Newton Fluid. 2011;166(19-20):1116-22. doi:DOI 10.1016/j.jnnfm.2011.06.007.
5. Tasoglu S, Peters JJ, Park SC, Verguet S, Katz DF, Szeri AJ. The effects of inhomogeneous boundary dilution on the coating flow of an anti-HIV microbicide vehicle. Phys Fluids. 2011;23(9):093101. doi:Artn 093101
Doi 10.1063/1.3633337.
6. Tasoglu S, Katz DF, Szeri AJ. Transient spreading and swelling behavior of a gel deploying an anti-HIV topical microbicide. J Nonnewton Fluid Mech. 2012;187-188:36-42. doi:10.1016/j.jnnfm.2012.08.008.
7. Mathworks. MATLAB. 7.11.0 ed. Natick, Massachusetts: MathWorks Inc.; 2010.
